# Impetigo herpetiformis during the puerperium triggered by secondary hypoparathyroidism: a case report

**DOI:** 10.1186/1757-1626-2-9338

**Published:** 2009-12-16

**Authors:** Usama M Fouda, Ragai M Fouda, Hussam M Ammar, Mohamed Salem, Mohamed EL Darouti

**Affiliations:** 1 Department of Obstetrics & Gynecology, Cairo University, Kasr Al Aini Street, Cairo, Egypt; 2 Department of Internal Medicine, Cairo University, Kasr Al Aini Street, Cairo, Egypt; 3 Department of Internal Medicine, University at Buffalo, New York, USA; 4 Department of Dermatology, Cairo University, Kasr Al Aini Street, Cairo, Egypt

## Abstract

A 38-year-old multiparous woman with post thyroidectomy hypoparathyroidism developed pruritic erythematous patches with multiple pustules on its margins on her thighs and groin accompanied by fever few days after delivery by caesarean section. Impetigo herpetiformis was diagnosed based on the typical clinicopathological findings. The patient was treated with intravenous fluids, calcium, Calcitrol and corticosteroids. The correction of hypocalcaemia was accompanied with rapid improvement of her skin disease and general condition. Our case is the fourth case of impetigo herpetiformis initially presented during puerperium and the first case of puerperal impetigo herpetiformis that is precipitated by secondary hypoparathyroidism. The awareness of the possible occurrence of impetigo herpetiformis during the puerperium allows early diagnosis, treatment and prevention of maternal complications.

## Case presentation

A 38-year-old Egyptian woman was admitted to Cairo University Hospital with extensive exfoliation of her skin. Two weeks prior to her admission, she delivered a healthy baby by uncomplicated caesarian section.

Few days after her delivery, she developed pruritic erythematous patches with multiple pustules on its margins on her thighs, groins and abdominal wall. She was treated empirically with hydrocortisone 1% cream. Few days later, the rash progressed and involved most of her body, she felt weak and developed chills. On examination, she appeared ill with a blood pressure of 90/60, pulse rate of 120/min, temperature of 38°C and respiratory rate of 20/min.

**Figure 1:**
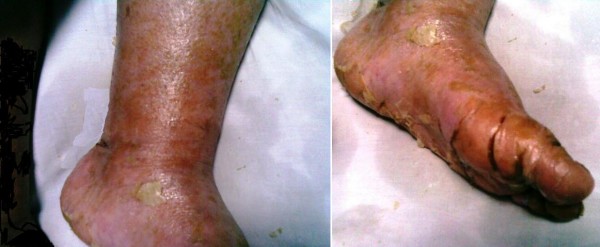
**Erythroderma, multiple crusted plaques and pustules in lower limb**.

She had erythroderma, multiple crusted plaques and extensive skin exfoliation of her trunk and extremities sparing her eyes and mucous membranes (Figure 1). There were no nails abnormalities noted. Chvostek and Trousseau signs were negative. There were no personal or family history of similar condition.

Thyroidectomy was performed for toxic nodular goiter during the second trimester. She developed secondary hypoparathyroidism after surgery. She was not compliant with her medications which included calcium, L-thyroxine and vitamin D.

Laboratory studies revealed leucocytic count of 21.500/ml (normal 4300-10800/ml) with 84% neutrophils (normal 45%-74%), total calcium 5.2 mg/dl (normal 8.5-10.5 mg/dl), phosphorus 5 mg/dl (normal 2.5-4.5 mg/dl), albumin 3.2 gm/dl (normal 3.5-5.5 gm/dl), blood urea nitrogen 64 mg/dl (normal 10-20 mg/dl), creatinine 1.4 mg/dl (normal < 1.5 mg/dl). Thyroid stimulating hormone 38.3 μU/ml (normal 0.4-0.6 μU/ml), thyroxine 0.39 Pmol/ml (normal 9-24 Pmol/ml) and parathormone 6.1 Pg/ml (normal 11-54 Pg/ml).

**Figure 2:**
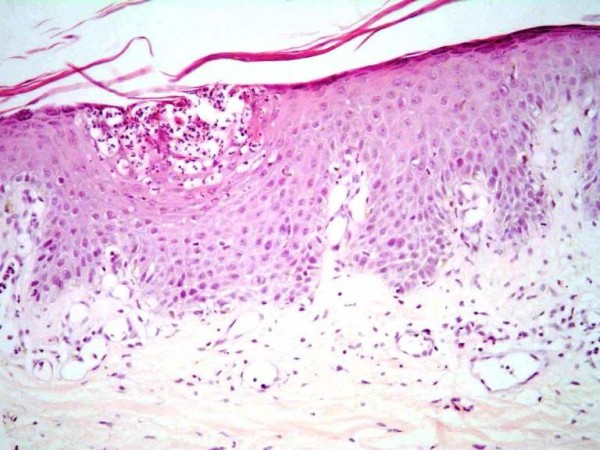
**Subcorneal spongiform pustules containing numerous neutrophils**.

Intravenous fluids, oral prednisone 60 mg/d, calcium gluconate 2 g/d, Calcitrol 0.25 μg/d, intravenous clindamycin 1200 mg/d and intravenous vancomycin 1 g/day were given. The correction of hypocalcaemia (within five days) was accompanied with rapid improvement of her skin disease and general condition, indicating the importance of correction of hypocalcaemia in patients with impetigo herpetiformis precipitated by hypoparathyroidism [1]. The skin biopsy was consistent with pustular psoriasis. (Figure 2)

**Figure 3:**
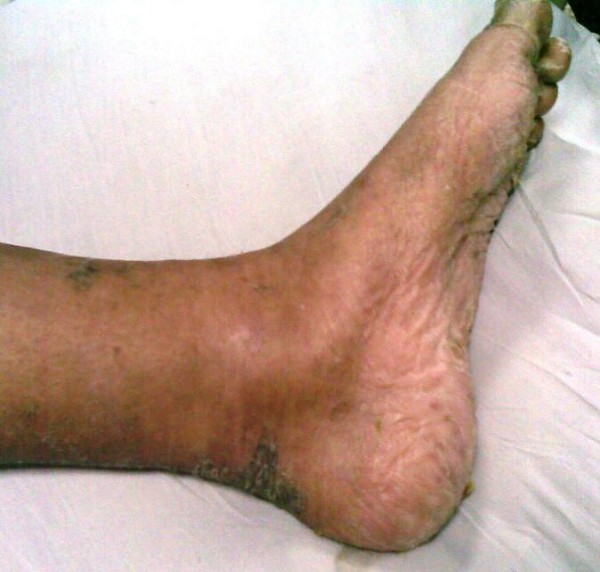
**Complete clearing of skin lesions after treatment**.

The patient was discharged from the hospital three weeks after her admission with near complete recovery of her skin (Figure 3).

Hebra introduced the term impetigo herpetiformis (IH) to described five pregnant women, all of them were in toxic condition, febrile and had extensive pustular eruption. Hebra classified it as a specific dermatosis of pregnancy [2]. IH usually present during the third trimester with a progressive course during pregnancy and then rapid resolution occurs during the puerperium. Few cases similar to our case were initially reported during the puerperium [3-5].

It is controversial, whether IH is a form of generalized pustular psoriasis based on the identical histopathology, or a distinct entity. The absence of personal and family history of psoriasis, the association with pregnancy and hypocalcaemia, its recurrence with subsequent pregnancies and the absence of development of chronic plaque psoriasis might suggest that IH is a distinct entity [1,6,7].

IH presents mainly by pustular eruption that begins symmetrically in the flexural and intertriginous areas and then extends centrifugally. The lesions are erythematous patches with sterile pustules at their margins. They form plaques and pustules that become eroded, then crusted. Generalized eruption and even exfoliation of the whole body can also occur as in our case. Mucous membranes can be also affected [6].

IH is usually associated with systemic features as fever, sweating, tachycardia, nausea, diarrhea, vomiting, lymphadenopathy, splenomegaly and tetany, in severe cases renal or cardiac failure may occur. Moreover, IH is associated with increased incidence of congenital anomalies and placental insufficiency resulting in increased risk of intrauterine growth retardation, stillbirth and neonatal deaths [7].

Although hypocalcemia in many cases of IH may be a secondary phenomenon due to extensive skin inflammation that result in extravasation of albumin and calcium bounded to it to the interstitial space [8]. It had been established that IH could be precipitated by hypoparathyroidism and hypocalcaemia, and could be cured by correction of hypocalcaemia in patients with hypoparathyroidism. Moreover there are several reports of IH occurring in men and non pregnant women with hypoparathyroidism and hypocalcaemia, suggesting the triggering role of hypocalcaemia in the disease process [1,8]. Katsambas et al suggested that the decrease of serum calcium (main decrease is in nonionized fraction with mild decrease in ionized calcium) and vitamin D during pregnancy and puerperium play a role in the development of IH during pregnancy and puerperium [3].

There are no guidelines for IH treatment. Fluid and electrolytes especially calcium should be monitored and normalized [1]. Corticosteroids are commonly used [6,7]. There are reports of successful treatment of IH during pregnancy with cyclosporine [9]. Other studies reported that Retinoids, methotrexate, psoralen and ultraviolet A (PUVA) can be used successfully during puerperium [6,7].

## Conclusion

According to the best of our knowledge this is the fourth case of impetigo herpetiformis initially presented during the puerperium and the first case of puerperal impetigo herpetiformis that is precipitated by secondary hypoparathyroidism. The awareness of the possible occurrence of IH during the puerperium allows early diagnosis, treatment and prevention of possible serious maternal complications.

## Abbreviations

IH: impetigo herpetiformis; PUVA: psoralen and ultraviolet A.

## Competing interests

The authors declare that they have no competing interests.

## Authors' contributions

All the authors analyzed and interpreted the patient data. UMF, RMF and HMA were involved in manuscript writing. M.E performed the histological examination of the skin lesions. All the authors approved the final manuscript.

## Consent

Written informed consent was obtained from the patient for publication of this case report and accompanying images. A copy of the written consent is available for review by the Editor-in-Chief of this journal.
